# Hypoxic Conditioned Medium from Human Amniotic Fluid-Derived Mesenchymal Stem Cells Accelerates Skin Wound Healing through TGF-β/SMAD2 and PI3K/Akt Pathways

**DOI:** 10.3390/ijms15010605

**Published:** 2014-01-06

**Authors:** Eun Kyoung Jun, Qiankun Zhang, Byung Sun Yoon, Jai-Hee Moon, Gilju Lee, Gyuman Park, Phil Jun Kang, Jung Han Lee, Areee Kim, Seungkwon You

**Affiliations:** 1 Laboratory of Cell Function Regulation, College of Life Sciences and Biotechnology, Korea University, Seoul 136-717, Korea; E-Mails: ek-young@hanmail.net (E.K.J.); qkzhang0915@gmail.com (Q.Z.); biosun302@korea.ac.kr (B.S.Y.); moonjaihi@hanmail.net (J.-H.M.); ggang1979@hanmail.net (G.L.); legnaym@korea.ac.kr (G.P.); rebeast@korea.ac.kr (P.J.K.); ano112@nate.com (J.H.L.); 2 Department of Pathology, College of Medicine, Korea University Guro Hospital, Seoul 152-703, Korea; E-Mail: ark@korea.ac.kr

**Keywords:** hypoxia, amniotic fluid-derived mesenchymal stem cells (AF-MSCs), wound healing, PI3K/AKT, TGF-β/SMAD2

## Abstract

In a previous study, we isolated human amniotic fluid (AF)-derived mesenchymal stem cells (AF-MSCs) and utilized normoxic conditioned medium (AF-MSC-norCM) which has been shown to accelerate cutaneous wound healing. Because hypoxia enhances the wound healing function of mesenchymal stem cell-conditioned medium (MSC-CM), it is interesting to explore the mechanism responsible for the enhancement of wound healing function. In this work, hypoxia not only increased the proliferation of AF-MSCs but also maintained their constitutive characteristics (surface marker expression and differentiation potentials). Notably, more paracrine factors, VEGF and TGF-β1, were secreted into hypoxic conditioned medium from AF-MSCs (AF-MSC-hypoCM) compared to AF-MSC-norCM. Moreover, AF-MSC-hypoCM enhanced the proliferation and migration of human dermal fibroblasts *in vitro*, and wound closure in a skin injury model, as compared to AF-MSC-norCM. However, the enhancement of migration of fibroblasts accelerated by AF-MSC-hypoCM was inhibited by SB505124 and LY294002, inhibitors of TGF-β/SMAD2 and PI3K/AKT, suggesting that AF-MSC-hypoCM-enhanced wound healing is mediated by the activation of TGF-β/SMAD2 and PI3K/AKT. Therefore, AF-MSC-hypoCM enhances wound healing through the increase of hypoxia-induced paracrine factors via activation of TGF-β/SMAD2 and PI3K/AKT pathways.

## 1. Introduction

Cutaneous wound healing encompasses a highly complex process involving an orderly regulated cascade of events of cells migration and proliferation, extracellular matrix (ECM) deposition, angiogenesis and remodeling [1–3]. In this complex process, fibroblasts interact with surrounding cells to produce the ECM, glycoproteins, adhesive molecules and various growth factors [4]. By cell-to-cell interactions, and cell-to-cytokine interdependencies, fibroblasts contribute to a variety of cell complexes, which not only repair wounds, but also maintain the integrity of skin [5].

Human amniotic fluid (AF) contains a very heterogeneous cell population which includes a variety of stem cells shed from embryonic and extra embryonic tissues during fetal development. Human mesenchymal stem cells (MSCs) are multipotent cells which are present in many tissues of the human body, including AF, and can form bone, cartilage, muscle or fat, as well as a variety of other connective tissues by differentiating into osteoblasts, chondrocytes, myoblasts or adipocytes, as well as various cells of other embryonic lineages [6–10]. They are an important source for regenerative medicine, such as in osteogenesis imperfect [11], bone fracture [12], myocardial infarction [13,14] and spinal injury [15]. AF-MSCs also exhibit similar characteristics and functional properties as other MSCs [16]. Moreover, recent evidence demonstrated that conditioned medium from bone marrow derived MSCs (BM-MSCs), adipose-derived MSCs (AD-MSCs) and AF-derived MSCs (AF-MSCs) enhanced the migration and proliferation of fibroblasts *in vitro* and accelerated wound healing in *in vivo* models [5,17–20]. These studies indicated not only that MSCs can reconstruct skin tissue, but that their conditioned medium also has the ability to promote the regeneration of skin tissue.

Oxygen is a potent biochemical signaling molecule which exerts significant effects on the growth and development of mammalian cells. The state of oxygen deficiency, hypoxia, is cell-type dependent, and affects critical cellular processes, such as proliferation [21], adhesion [22], apoptosis [23], metabolism [24], ECM secretion [25], growth factor expression [26], and differentiation patterns [27]. Hypoxia can lead to apoptosis, but hypoxic preconditioning of MSCs can reduce hypoxia-induced cell death, which is caused by the paracrine activity of MSCs inducing the upregulation of various secretable factors, such as vascular endothelial growth factor (VEGF), transforming growth factor beta 1 (TGF-β1) and others [20,28]. It has been demonstrated that the conditioned medium of AD-MSCs harvested under hypoxic conditioned medium (hypoCM) significantly promoted the migration of human dermal fibroblasts, and obviously reduced the wound area in an *in vivo* model, compared with those in normoxic conditioned medium (norCM) [20]. However, little is known regarding the underlying mechanisms involved in hypoCM-induced migration and proliferation of fibroblasts, which are important in accelerating wound healing.

This study demonstrated that hypoxia enhanced the secretion of paracrine factors from AF-MSCs related with proliferation and survival of cells. Moreover, we also determined that hypoxic conditioned medium from AF-MSCs (AF-MSC-hypoCM) enhanced dermal fibroblasts migration *in vitro* and wound healing *in vivo* by TGF-β/SMAD2 and PI3K/AKT pathways.

## 2. Results

### 2.1. Hypoxia Promotes Proliferation and Survival of AF-MSCs

**Figure 1.**
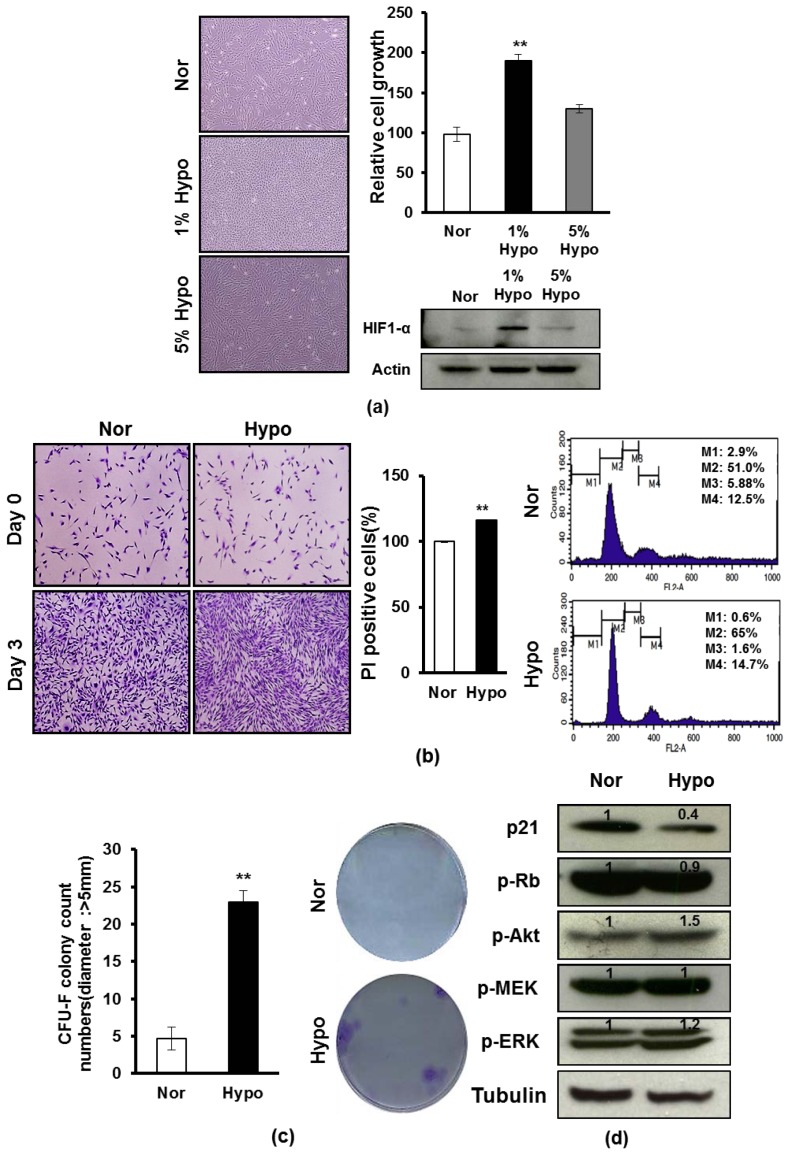
The Effect of hypoxia on the proliferation and survival of AF-MSCs. (**a**) AF-MSCs were cultured under normoxic or hypoxic conditions (1% or 5% O_2_) after 3 days, showing different growth and expressing different protein levels of HIF1-α at protein levels. All cells were stained by 0.01% crystal violet. The graph shows the relative cell growth; (**b**) PI-stained AF-MSCs that were cultured under normoxic or hypoxic condition (1% O_2_) after 3 days, showing the increase of the number of PI-stained cells in the G1phase of cell cycle in responses to hypoxic condition. (M1: apoptotic cells; M2: G1; M3: S; M4: G2/M); (**c**) CFU-assay of AF-MSCs cultured under normoxic and hypoxic condition showed that the clonogenic capacity of AF-MSCs increased under hypoxic condition compared to normoxic condition; and (**d**) AF-MSCs under hypoxic condition express different protein levels of cell proliferation- or survival-related regulators (P21, p-Rb, p-Akt, p-MEK and p-ERK). Data are expressed as the mean ± SD. ** *p* < 0.01.

To investigate whether hypoxia influences the proliferation of AF-MSCs, we examined the proliferation of AF-MSCs cultured under either normoxia (20% O_2_, 5% CO_2_) or hypoxia (1% or 5% O_2_) for 3 days. When cultured in 1% O_2_ hypoxia, the expansion level of AF-MSCs was higher compared to when cultured in 5% O_2_ hypoxia or normoxia (Figure 1a). Likewise, we also examined the protein levels of hypoxia inducible transcription factor 1α (HIF-1α) under the same conditions, showing that its expression was significantly increased under 1% O_2_ hypoxic condition (Figure 1a). We next tested the effect of hypoxia on the survival and proliferation of AF-MSCs, showing the number of viable AF-MSCs was significantly increased under 1% O_2_ hypoxic condition compared to normoxic condition, and also showing the cell numbers in the G_1_ phase (65% *vs.* 51%) of cell cycle was increased (Figure 1b). To compare the potentials of proliferation and clonogenic capacity of AF-MSCs under normoxic and 1% hypoxic conditions, a CFU-F assay was conducted and the colonies with a diameter >5 mm were counted [19,29]. As shown in Figure 1c, hypoxic condition promoted the relative clonogenecity of AF-MSCs. The results showed that at seven days of culture, 4.7 ± 1.5/100 cells/cm^2^ colonies were formed from hypoxia-treated AF-MSCs, whereas 23 ± 1.7/100 cells/cm^2^ c colonies were formed from normoxia-treated AF-MSCs (Figure 1c). Due to the close relationship among cell proliferation and cell cycle, we further examined the protein levels of cell cycle regulators in AF-MSCs that were cultured in normoxia or 1% O_2_ hypoxia condition, and found that p21 and the phosphorylation of Rb were downregulated, and also observed increased phosphorylation of AKT, MEK and ERK, which were found to be important during cell proliferation and survival responses to 1% O_2_ hypoxia (Figure 1d). The results suggest that 1% hypoxia enhances the proliferation and survival of AF-MSCs via modulation of the expression of cell cycle regulators.

### 2.2. Hypoxia Maintenances Mesenchymal Differentiation Potentials

**Figure 2.**
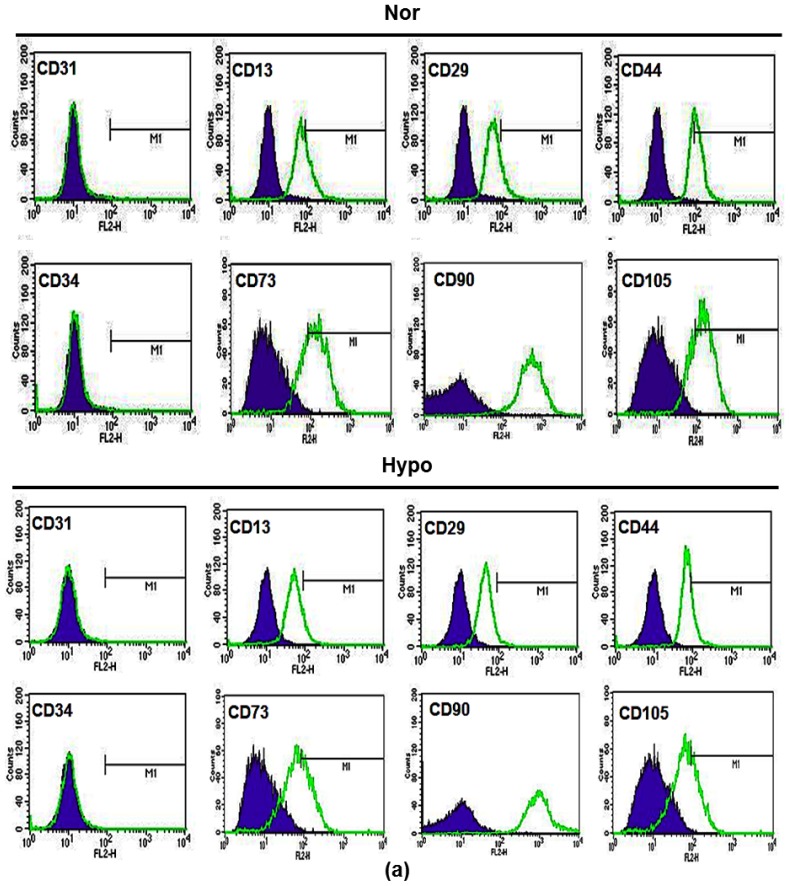

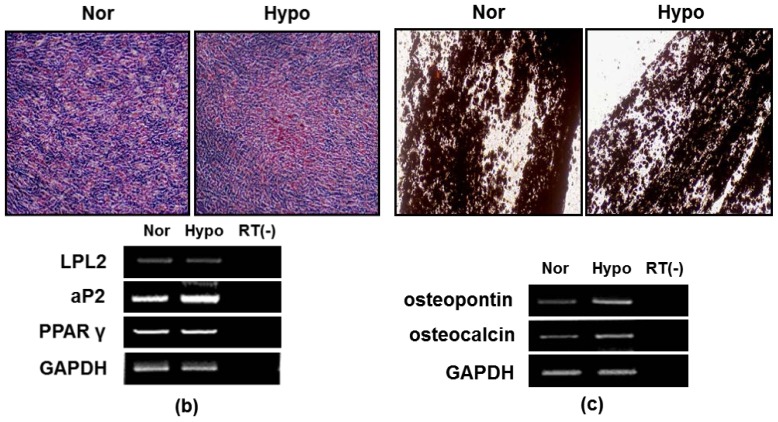
The effect of hypoxia on the characterization of AF-MSCs. (**a**) FACS analysis of AF-MSCs that were cultured under normoxic or hypoxic condition show similar expression of the typical MSC marker proteins CD13, CD29, CD44 and CD90, and not expression of the endothelial cell markers (CD31 and CD34); (**b**) AF-MSCs that were cultured under both normoxic and hypoxic condition differentiate to adipocyte-like cells in adipogenic induction medium after 2 weeks, which were demonstrated by Oil red O staining and the mRNA expression of adipogenic markers (LPL2, aP2 and PPARγ2); and (**c**) AF-MSCs that were cultured under both normoxic and hypoxic condition differentiate to osteoblasts-like cells in osteogenic induction medium after 3 weeks, which were demonstrated by alizarin red S staining and the mRNA expression of osteogenic markers (osteopontin and osteocalcin).

We next investigated whether hypoxia influences the constitutive characteristics of AF-MSCs. We examined multiple MSC makers of AF-MSCs which were cultured in either normoxic or 1% O_2_ hypoxic culture conditions. FACS analysis demonstrated that >95% of AF-MSCs which were cultured in either normoxia or hypoxia expressed the typical MSC marker proteins CD13, CD29, CD44, CD73, CD90 and CD105, and did not express the endothelial cell markers (CD31 and CD34) as the isotype control (Figure 2a). Because normal MSCs can generally differentiate into adipocyte and osteocytes in differentiated media [30–32], we further examined the effect of hypoxia on the differentiation potentials of AF-MSCs. AF-MSCs that were cultured in normoxic and hypoxic conditions were subjected to adipogenic differentiation [29,33]. After 2 weeks, the differentiated AF-MSCs in responses to either normoxia or hypoxia exhibited similar levels of small oil droplets in the cytoplasm, as revealed by Oil Red O staining (Figure 2b). RT-PCR analysis of adipogenic genes expression also revealed similar degrees of the upregulation of lipoprotein lipase (LPL), adipocyte fatty acid-binding protein 2 (aP2) and peroxisome proliferator-activated receptor γ2 (PPAR γ2) (Figure 2c). In the same way, AF-MSCs which were cultured in AF-MSC-norCM and AF-MSC-hypoCM were subjected to osteogenic differentiation for 2–3 weeks, exhibiting similar levels of Alizarin red staining (Figure 2c). RT-PCR analysis of osteogenic gene expression also revealed similar levels of upregulation of osteopontin and osteocalcin (Figure 2c). The data demonstrated that hypoxia maintains the basic characterization of AF-MSCs, showing specific surface marker expression and differentiation potentials, as BM-MSCs or AD-MSCs.

### 2.3. Hypoxia Facilitates the Secretion of Paracrine Factors of AF-MSCs and Hypoxic Conditioned Medium Accelerates the Proliferation and Migration of Human Dermal Fibroblasts

**Figure 3.**
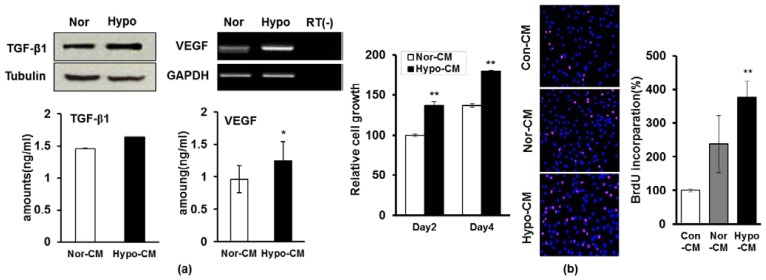

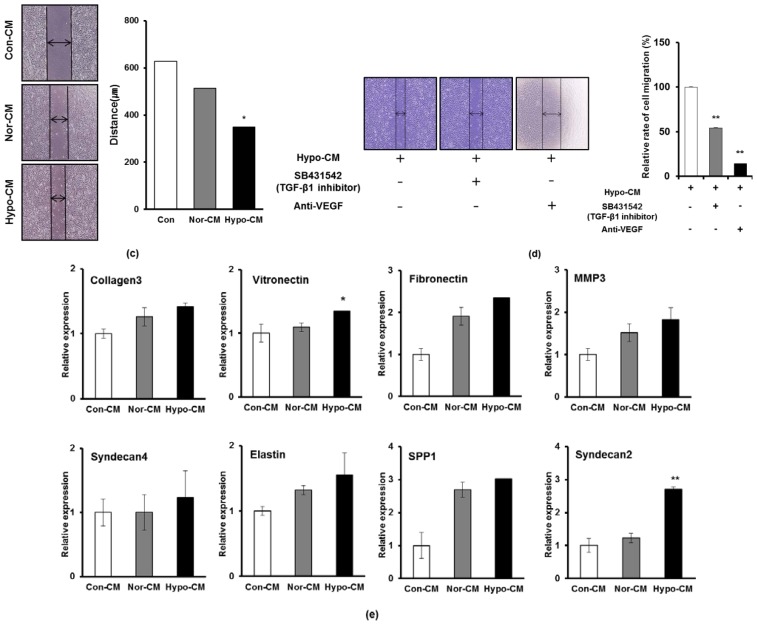
The effect of AF-MSC-hypoCM with paracrine factors on the proliferation and migration of human dermal fibroblast. (**a**) RT-PCR, western blot and ELISA assay was performed to determine the expression levels of paracrine factors (TGF-β1 and VEGF) from cells (AF-MSCs cultured under normoxic and hypoxic condition) and their conditioned media (AF-MSC-norCM and AF-MSC-hypoCM), showing the expression levels of TGF-β1 and VEGF in either cells or condition medium were increased in response to hypoxic condition; (**b**) The number of viable dermal fibroblasts was significantly increased when cultured in AF-MSC-hypoCM compared with in AF-MSC-norCM, indicated that AF-MSC-hypoCM causes the increases of DNA replication of fibroblasts, which was demonstrated by BrdU assay; (**c**) Micrographs of scratch-wound-closure assays following either AF-MSC-norCM or AF-MSC-hypoCM treatment. Dermal fibroblasts were synchronized by serum starvation, wounded, and treated with either AF-MSC-norCM or AF-MSC-hypoCM treatment for 24 h before being photographed. A significant increase in the migration of human dermal fibroblasts into the wound area was observed in response to AF-MSC-hypoCM treatment at 18 h after scratch injury. Arrows indicate the distance of wound, showing the decrease in AF-MSC-hypoCM treatment; (**d**) Effect of inhibition of TGF-β1 and VEGF on AF-MSC-hypoCM-induced migration of cells. AF-MSC-hypoCM-enhanced scratch-wound-closure and cell migration were significantly weakened by the inhibitors of TGF-β1 and VEGF, SB431542 and anti-VEGF, respectively; and (**e**) Quantitation of changes in gene expression of ECM molecules detected by real-time PCR after either AF-MSC-norCM or AF-MSC-hypoCM treatment. Data are expressed as the mean ± SD. * *p* < 0.05, ** *p* < 0.01.

As it was demonstrated that hypoxia can upregulate the paracrine activity of BM-MSCs or AD-MSCs [20,28], we next investigated whether hypoxia can influence the secretion of paracrine factors from AF-MSCs. RT-PCR, western blot and ELISA analyses of either AF-MSCs cultured under hypoxic condition or AF-MSC-hypoCM revealed enhanced expression of VEGF and TGF-β1 compared to either cells cultured under normoxic condition or AF-MSC-norCM (Figure 3a). Moreover, gene assay was also performed to detect the different expression of global mRNA among AF-MSCs cultured under hypoxic and normoxic condition (Table 1 and Supplementary Figure S1), which revealed the expression of VEGF was increased in AF-MSCs cultured in hypoxic condition compared to normoxic condition.Next, we investigated whether AF-MSC-hypoCM influences the proliferation of human dermal fibroblasts. The number of viable dermal fibroblasts was significantly increased when cultured in AF-MSC-hypoCM compared with those cultured in AF-MSC-norCM (Figure 3b). Moreover, we further tested whether the increased proliferation of dermal fibroblasts resulted from increased cell-cycle progression using BrdU staining, which showed that the absolute number of BrdU-positive cells was higher in AF-MSC-hypoCM compared with AF-MSC-norCM (Figure 3b).

Moreover, we investigated whether AF-MSC-hypoCM influences the migration of human dermal fibroblasts, which was examined by the standard “scratch test” technique [34,35]. Quantification of migrated cells revealed significantly faster migration under cultivation of AF-MSC-hypoCM compared with AF-MSC-norCM in an artificial wound model (Figure 3c). Likewise, we also found that the inhibitors of VEGF and TGF-β1 significantly weakened the AF-MSC-hypoCM-enhanced migration of cells (Figure 3d). Furthermore, the real-time PCR assay revealed that the expression of ECM molecules which are involved in cell migration, including Collagen type III, vitronectin, fibronectin, MMP1, Syndecan 2, Syndecan 4, SPP 1, and Elastin, were increased under AF-MSC-hypoCM compared to AF-MSC-norCM (Figure 3e). These findings indicate that AF-MSC-hypoCM more efficiently enhances wound healing events, such as proliferation and migration of dermal fibroblasts, as well as collagen production.

### 2.4. AF-MSCs Hypoxic Conditioned Medium Regulates TGF-β/SMAD2 and PI3K/AKT Pathway in Human Dermal Fibroblast

**Figure 4.**
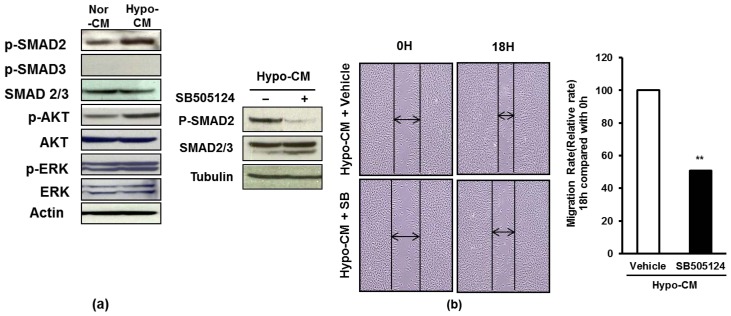

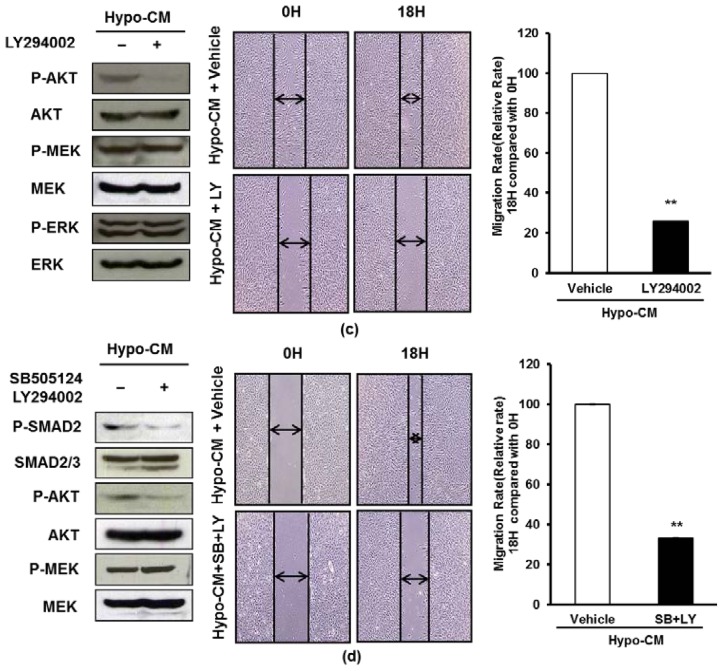
AF-MSC-hypoCM influences the migration of human dermal fibroblasts through regulating TGF-β/SMAD2 and PI3K/AKT signal pathways. (**a**) Western blot assay was performed to determine the protein levels of phosphorylated SMAD2/3, AKT and ERK in human dermal fibroblasts that were cultured in either AF-MSC-norCM or AF-MSC-hypoCM for 18 h, showing that the expression of phosphorylated SMAD2 and AKT were increased in response to AF-MSC-hypoCM; (**b**) Human dermal fibroblasts were cultured for 18 h in the presence or absence of TGF-β inhibitor, SB505124. The protein levels of phosphorylated SMAD2 were decreased in response to AF-MSC-hypoCM. A significant increase in the migration of human dermal fibroblasts into the wound area was observed in response to AF-MSC-hypoCM treatment at 18 h after scratch injury. Arrows indicate the distance of wound, showing the decrease in AF-MSC-hypoCM treatment; (**c**) Human dermal fibroblasts were cultured for 18 h in the presence or absence of PI3K inhibitor, LY294002. The protein levels of phosphorylated AKT were decreased in response to AF-MSC-hypoCM. A significant increase in the migration of human dermal fibroblasts into the wound area was observed in response to AF-MSC-hypoCM treatment at 18 h after scratch injury. Arrows indicate the distance of wound, showing the decrease in AF-MSC-hypoCM treatment; and (**d**) Human dermal fibroblasts were cultured for 18 h in the presence or absence of both SB505124 and LY294002. The protein levels of phosphorylated SMAD2 and AKT were decreased in response to AF-MSC-hypoCM. A significant increase in the migration of human dermal fibroblasts into the wound area was observed in response to AF-MSC-hypoCM treatment at 18 h after scratch injury. Arrows indicate the distance of wound, showing the decrease in AF-MSC-hypoCM treatment. Data are expressed as the mean ± SD. ** *p* < 0.01.

To investigate the molecular mechanisms of this enhanced proliferation and migration of dermal fibroblasts upon stimulation by AF-MSC-hypoCM, we examined TGF-β/SMAD2, PI3K/AKT and ERK signal pathways, which are activated during wound healing [19,36]. The dermal fibroblasts cultured in AF-MSC-hypoCM expressed higher levels of phosphorylated SMAD2 and AKT compared to in AF-MSC-norCM (Figure 4a). Moreover, we further tested whether the TGF-β/SMAD and PI3K/AKT signal pathways influence the migration of dermal fibroblasts cultured in AF-MSC-hypoCM. TGF-β inhibitor, SB505124, resulted in a significant reduction of phosphorylated SMAD2 and inhibited the migration of fibroblasts stimulated by AF-MSC-hypoCM (Figure 4b). Likewise, PI3K inhibitor, LY294002, also resulted in a remarkable decrease of phosphorylated AKT, and suppressed the migration of fibroblasts stimulated by AF-MSC-hypoCM (Figure 4c). Furthermore, treatment of both SB505124 and LY294002 resulted in the same outcome (Figure 4d). The results suggest that AF-MSC-hypoCM regulated the migration of dermal fibroblasts via both the TGF-β/SMAD2 and PI3K/AKT signal pathways.

### 2.5. AF-MSCs Hypoxic Conditioned Medium Accelerates Wound Healing *in Vivo*

**Figure 5.**
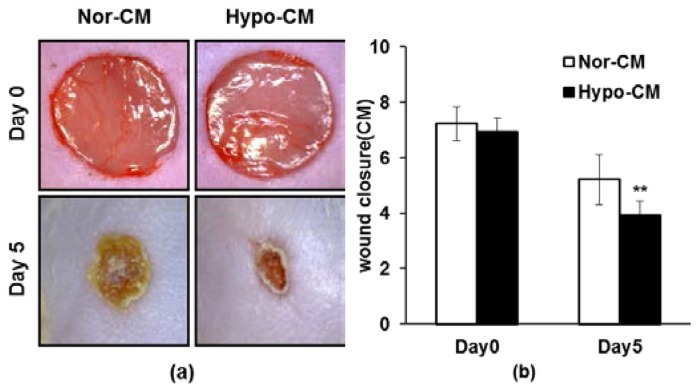

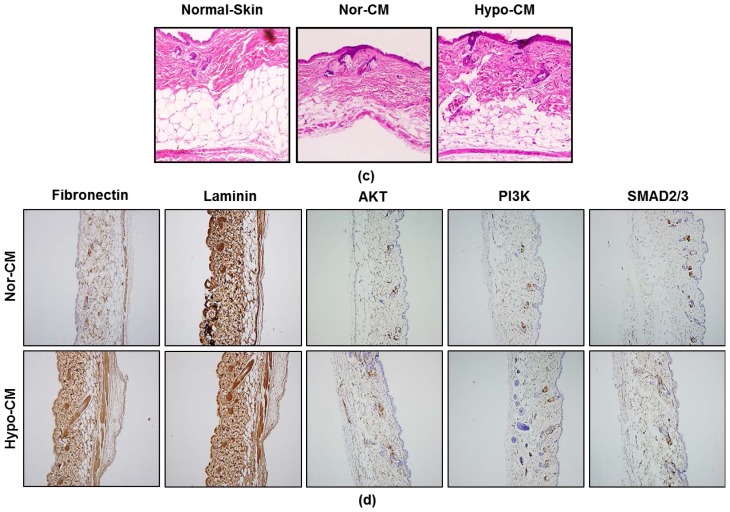
The effect of AF-MSC-hypoCM on wound closure. (**a**) Representative images of wounds after 5 days of treatment, showing that the wound sizes were observably decreased in response to AF-MSC-hypoCM; (**b**) Measurement of wound sizes treated with AF-MSC-norCM and AF-MSC-hypoCM, respectively, showing that wound closure was significantly increased in response to AF-MSC-hypoCM at day 5; (**c**) Histomorphometric analysis of wound closure showed that AF-MSC-hypoCM treated mice revealed significant improvement compared to AF-MSC-norCM treated mice at day 20; and (**d**) Immunohistochemistric analysis of wounded skin showed that AF-MSC-hypoCM treatment increased obviously the expression of ECM marker (fibronectin) and AKT, PI3K and SMAD2/3 proteins. Representative data from 10 animals per group are shown. Data are expressed as the mean ± SD. ** *p* < 0.01.

To investigate whether AF-MSC-hypoCM influences wound healing *in vivo*, we topically applied 100 μL of concentrated AF-MSC-hypoCM to excisional wounds created in ICR mice [17,37]. Similar wounds treated with vehicle medium (DMEM/F12) and AF-MSC-norCM were used as controls, respectively. Careful measurement of the wounds at five days indicated that AF-MSC-hypoCM significantly accelerated wound closure compared to DMEM/F12 or AF-MSC-norCM (Figure 5a). In addition, the initiation of re-epithelialization from the wound edge was more clearly observed in the wounds treated with AF-MSC-hypoCM. The degree of wound closure in controls and AF-MSC-hypoCM-treated mice was quantitatively measured in Figure 5b, showing an increased degree of wound closure responses to AF-MSC-hypoCM. H&E staining of wounded skin was performed at 20 days after surgery, which showed that the skin structure of wounds treated by AF-MSC-hypoCM was more similar to normal skin structure compared to DMEM/F12 or AF-MSC-norCM (Figure 5c). Moreover, the DAB staining of wounded skin was also performed, which showed that the expression of ECM marker, fibronectin, was obviously increased after AF-MSC-hypoCM treatment, which also showed the expression of AKT, PI3K and SMAD2/3 proteins were increased after AF-MSC-hypoCM treatment. The results show that AF-MSC-hypoCM improves the wound-healing effect *in vivo* through fibronectin-enhanced cell migration and both the TGF-β/SMAD2 and PI3K/AKT signal pathways.

## 3. Discussion

Our previous studies demonstrated that AF-MSCs secreted high levels of cytokines, growth factors and chemokines, which could enhance wound healing. Furthermore, normoxic conditioned medium from AF-MSCs (AF-MSC-norCM) improved the effectiveness of tissue repair [19]. Our present work reveals that hypoxic conditioned medium of AF-MSCs (AF-MSC-hypoCM) results in the increased proliferation and migration of human dermal fibroblasts *in vitro*, and the wound healing *in vivo*. In AF-MSC-hypoCM, specific growth factors involved in wound healing (TGF-β and VEGF) were increased. Moreover, the effects of AF-MSC-hypoCM on the proliferation and migration of dermal fibroblasts were significantly increased, and these effects were reversed by neutralizing inhibitors against TGF-β/SMAD2 and PI3K/AKT signal pathways. AF-MSC-hypoCM obviously enhanced the wound closure *in vivo*, and increased the expression of fibronectin, AKT, PI3K and SMAD2 involved in proliferation and migration of cells. These results demonstrate that hypoxia enhanced the wound-healing function of AF-MSC-norCM through the upregulation of growth factors involved in TGF-β/SMAD2 and PI3K/AKT signal pathways.

AF is an abundant source of MSCs which exhibit a phenotype and multi-lineage differentiation potential similar to that of BM-derived MSCs [16,30–32]. MSCs have great potential in the fields of tissue engineering and regenerative medicine [38]. Recent studies have focused on the effect of reducing oxygen tension (hypoxia) on the behavior and functions of MSCs. MSCs under hypoxic condition responds to transcription factors, most notably hypoxia inducible factor 1α (HIF-1α). HIF-1α could induce the expression of the glucose-6-phosphate transporter which upregulates gluconeogenesis. The increased availability of glucose through upregulated gluconeogenesis would help extend the survival of MSCs under hypoxic condition (<2% O_2_) [39]. Moreover, HIF-1α also enhanced the proliferation of MSCs by regulating expression of cell-cycle regulators, such as p21, p27, p53 and p-Rb [40,41]. It is known that p21 mediates the G1 arrest of cell cycle, and its inhibition can activate the G1 checkpoints of cell cycle, which enhance the proliferation of cells. The cells cultured under hypoxic condition activated the PI3K/Akt and MEK/ERK signaling pathways, while enhancing their viability, proliferation and migration [42–45]. Consistent with these studies, our experimental findings demonstrated that hypoxia enhanced not only expression of HIF-1α, but also the proliferation and survival of AF-MSCs through regulation of cell-cycle regulator p21 and the PI3K/Akt and MEK/ERK signaling pathways. Moreover, hypoxia did not significantly change the stemness of AF-MSCs, showing the expression of MSCs specific markers, such as CD13, CD29, CD44, CD73, CD90 and CD105, and the adipogenic and osteogenic differentiation of AF-MSCs. These results suggest that hypoxia not only enhances the proliferation, survival and clonogenic capacity of AF-MSCs, but also maintains the potential of AF-MSCs.

MSCs can play an important role in various processes via the secretion of trophic factors. In our previous studies, AF-MSC-norCM significantly improved the proliferation and migration of dermal fibroblasts and enhanced the wound-healing process in a skin injury model [19]. We, and others, have demonstrated that hypoxic conditioned medium from MSCs enhanced the proliferation and migration, as well as collagen production of dermal fibroblasts [20], and improved the efficiency of wound healing in a mouse model of skin injury. It has been previously demonstrated that human MSCs cultured under hypoxia exhibited an increased protein release in medium for such proteins as VEGF, FGF2, TGF-β1 and IL6, which are involved in the inflammatory phase of wound healing [3,19,46]. Indeed, we also found that the expression of VEGF and TGF-β1 at a protein level were upregulated in AF-MSC-hypoCM. Furthermore, it has also been shown that VEGF and TGF-β1 can induce the proliferation and collagen expression of fibroblasts [47,48]. There is also evidence that VEGF and TGF-β1 regulate PI3K/Akt and TGF-β/SMAD2 pathways [36,49]. To investigate the relationship of PI3K/Akt or TGF-β/SMAD2 pathways and wound healing, PI3K inhibitor LY294002 and TGF-β inhibitor SB505124 were used to examine the effects of AF-MSC-hypoCM on *in vitro* wound healing (migration of fibroblasts), showing that inhibition of either PI3K/Akt or TGF-β/SMAD2 pathways reduced the migration of fibroblasts which were enhanced by VEGF and TGF-β1. Moreover, AF-MSC-hypoCM not only improved the wound closure but also induced the expression of ECM marker (fibronectin), AKT, PI3K and SMAD2/3 proteins in wounded skin *in vivo*, which involved in cell proliferation and migration. These results suggest that AF-MSC-hypoCM, containing growth factors, enhances the proliferation and migration of dermal fibroblasts via PI3K/Akt and TGF-β/SMAD2 pathways, and accelerates wound healing.

## 4. Experimental Section

### 4.1. Cell Culture

Informed consent was obtained from all subjects, and all studies were conducted with strict adherence to the guidelines of the Institutional Review Board of Korea University, Seoul, Korea. Amniotic fluid (AF) was obtained by amniocentesis performed for fetal karyotyping between 16- and 20-weeks of gestation. AF-derived cells were cultured from AF as previously described, or were obtained from confluent back-up human amniocentesis cultures from a clinical cytogenetics laboratory [6]. Briefly, primary cell cultures were established in α-MEM medium (Grand Island, NY, USA) containing 15% ES-FBS (ES qualified-fetal bovine serum), 1% glutamine, and 1% penicillin/streptomycin (Gibco/Invitrogen, Carlsbad, CA, USA), supplemented with 18% Chang B and 2% Chang C (Irvine Scientific, Santa Ana, CA, USA) at 37 °C in a 5% CO_2_ atmosphere. At this stage, a mixture of two morphologically distinct groups attached and formed colonies. For the selective culture of AF-derived mesenchymal stem cells (AF-MSCs), cells were harvested by trypsinization and were then plated in low-glucose DMEM medium (Gibco/Invitrogen) supplemented with 10% fetal bovine serum (FBS; Hyclone, Logan, UT, USA) and 4 ng/mL basic fibroblast growth factor (FGF2; R&D Systems, Minneapolis, MN, USA), and incubated as described previously. A morphologically homogeneous population of AF-MSCs was obtained after 2 rounds of subculture. These AF-MSCs were maintained in a humidified atmosphere in an incubator under 5% CO_2_ at 37 °C. The clonal AF-MSCs were subcultured routinely at a dilution of 1:3 and were not permitted to progress beyond ~70% confluence. Cells were then tested for genetic markers, cellular surface antigens, and differentiation potentials. Human dermal fibroblasts were purchased and cultured according to the manufacturer’s instructions (ATCC, Manassas, VA, USA).

### 4.2. Survival and Proliferation of AF-MSCs

The viability of the cells was measured using propidium iodide (PI) staining in triplicate. AF-MSCs were grown to near confluence under normoxia (20% O_2_, 5% CO_2_) and hypoxia (1% or 5% O_2_, 20% CO_2_). After trypsinization, AF-MSCs were washed twice with DPBS, fixed with absolute methanol, and stained with the PI solution (25 μg/mL). The positive staining population was quantified by direct fluorescence using FACS analysis.

Cell growth was determined by plating fibroblasts at a density of 5 × 10^4^ cells/well (24-well plates) and synchronizing them in triplicate in proliferation medium [34]. AF-MSCs were incubated under normoxia (20% O_2_, 5% CO_2_) and hypoxia (1% or 5% O_2_, 20% CO_2_) for 72 h. Cells were then fixed with 10% formalin and stained with 0.01% crystal violet solution. Crystal violet solution from stained cells was extracted using 10% acetic acid and was subjected to spectrophotometric analysis (600 nm) to determine the relative cell growth rates.

### 4.3. Colony-Forming Unit Fibroblast (CFU-F) Assay

The CFU-F assay was performed by first plating AF-MSCs at a density of 100 cells/well in 6-well culture dishes (BD Biosciences, San Jose, CA, USA) prior to culture under normoxic or hypoxic conditions in triplicate, respectively. After 14 days of culture, cells were washed twice with PBS and were fixed with 10% formalin for 20 min at room temperature. To visualize and enumerate CFUs, the cells were stained with 0.01% crystal violet solution for 20 min at room temperature, and were then washed with deionized water and air dried. CFU-colonies were typically between 5 and 8 mm in diameter, and were scored macroscopically [19,29].

### 4.4. Preparation of Conditioned Media from Normoxia (AF-MSC-norCM) or Hypoxia-Treated AF-MSCs (AF-MSC-hypoCM)

AF-MSCs were plated at a concentration of 5 × 10^5^ cells/100-mm plate and were then incubated in proliferation medium overnight. The attached cells were washed three times with phosphate-buffered saline (PBS), and the medium was replaced with serum-free DMEM/F12 in order to generate conditioned medium (CM) which was serum-free and compatible for culture of AF-MSCs. The CM was prepared by incubating the cells under normoxia (20% O_2_, 5% CO_2_) and hypoxia (1% or 5% O_2_, 20% CO_2_) for 72 h. Both CMs were then collected, centrifuged at 1000 rpm for five min, and filtered through a 0.20-μm syringe filter. For *in vivo* experiments, Both CMs were further concentrated (5×) by ultrafiltration using centrifugal filter units with a 5-kDa cutoff (Millipore Corporation, Bedford, MA, USA) following the manufacturer’s instructions.

### 4.5. Adipogenic and Osteogenic Differentiation

Differentiation of each sample was performed according to the previously described protocol [29,33]. Briefly, the cells were seeded at a density of 1.5 × 10^4^ cells/well in six-well culture dishes, and were cultured in high-glucose DMEM with 10% FBS until they reached 100% confluence. They were then subjected to three cycles of induction/maintenance by sequentially culturing the cells in adipogenic induction medium [high-glucose DMEM (Invitrogen, Carlsbad, CA, USA) supplemented with 1 mM dexamethasone (Sigma-Aldrich, St. Louis, MO, USA), 0.5 mM 3-isobutyl-1-methyl-xanthine (Sigma-Aldrich), 10 ng/mL recombinant human insulin (Sigma-Aldrich), 100 mM indomethacin (Sigma-Aldrich), and 10% FBS] for seven days, adipogenic maintenance medium (high-glucose DMEM supplemented with 10 ng/mL recombinant human insulin and 10% FBS) for 14 days, and control medium (high-glucose DMEM supplemented with 10% FBS) for seven days; this process was repeated three times. After differentiation, the cells were fixed with 10% formalin (Sigma-Aldrich), washed, and stained with 2% (*w*/*v*) Oil Red O reagent (Sigma-Aldrich) for 5 min at room temperature to examine the generation of oil droplets in the cytoplasm.

Differentiation of each sample was performed according to the previously described protocol [29,33]. Briefly, cells were seeded at a density of 3 × 10^3^ cells/cm^2^ in six-well culture dishes (BD Biosciences), cultured in high-glucose DMEM (Gibco/Invitrogen) with 10% FBS until they reached 70%–80% confluence, and then fed twice a week for 2.5 weeks with osteogenic induction medium [IMDM basal medium (Gibco/Invitrogen) supplemented with 100 nM dexamethasone, 10 mM β-glycerophosphate, 0.2 mM ascorbate, and 10% FBS] and control medium (IMDM basal medium supplemented with 10% FBS). Osteogenic differentiation was detected by fixing the cells with 10% formalin (Sigma-Aldrich) for 15 min at room temperature and then staining them with Alizarin red S [29,50].

### 4.6. BrdU Assay

Cells were seeded in four-well plates, and after serum starvation, cells were cultured under normoxia and hypoxia in BrdU (10 μM; Sigma-Aldrich) in triplicate, respectively. After 24 h, cells were fixed and stained for BrdU incorporation by immunostaining using a BrdU staining kit (Invitrogen). The BrdU label index, defined as the proportion of total cells incorporating BrdU into the nucleus, was determined by counting BrdU-immunolabeled cells over total cells under phase contrast. The percentage of BrdU incorporation was calculated according to the following formula: % of BrdU-positive cells = (number of BrdU-positive cells/number of cells) × 100%. The data from three independent experiments were averaged, and the mean and standard deviation are shown [51,52].

### 4.7. RT-PCR and Quantitative Real Time Polymerase Chain Reaction

RNA in triplicate were prepared from samples using TRIzol according to the manufacturer’s instructions (Invitrogen), and cDNA was generated using Reverse Transcriptase II (Invitrogen) according to the manufacturer’s instructions. To amplify various marker genes, 25 ng cDNA was used along with the PCR primers (Bioneer, Daejeon, Korea) under the conditions outlined in Supplementary Table S1. Reaction mixtures (20 μL) were prepared as described above. The amplification program consisted of 24–35 cycles of the following parameters: 94 °C for 30 s; annealing at 62 °C for 30 s, and extension at 72 °C for 30 s, followed by a final amplification step for 10 min at 72 °C. We confirmed that the levels of the different PCR targets generated by 24–35 PCR cycles were in the linear range [53]. Real-time RT-PCR was conducted using the iCycler IQ (Bio-Rad, Hercules, CA, USA). Reactions were performed using SYBR-Green PCR Master Mix (Bio-Rad). As an internal control, levels of glyceraldehyde-3-phosphatedehydrogenase (GAPDH) were quantified in parallel with target genes. Normalization and fold changes were calculated using the ΔΔ*Ct* method [54].

### 4.8. Western Blot

To detect protein expression in response to AF-MSC-norCM or AF-MSC-hypoCM, cells were plated at a density of 3 × 10^5^ cells/100-mm plate, allowed to attach for 12 h, and cultured with serum-free DMEM for 48 h. Cells were then treated with AF-MSC-norCM or AF-MSC-hypoCM for the indicated times (30 min–8 h). To detect protein expression and modification in response to treatment with the transforming growth factor beta (TGF-β) inhibitor and phosphatidylinositol 3-kinase (PI3K) inhibitor, SB505124 and LY294002 (Sigma-Aldrich), cells were plated at a density of 7 × 10^5^ cells/100-mm plate and were cultured for the indicated times (30 min) in AF-MSC-hypoCM in the presence or absence of the inhibitors. Total protein was extracted with RIPA buffer. Lysates were then centrifuged at 12,000× *g* for 30 min at 4 °C. Protein concentrations were determined using the Bradford assay kit (Bio-Rad, Hercules, CA, USA). Proteins were separated using precast 4%–12% gradient SDS-PAGE (Invitrogen) and were transferred onto polyvinylidene difluoride membranes (Millipore, Bedford, MA, USA). Blots were incubated with the indicated primary antibodies at 4 °C and horseradish peroxidase-conjugated anti-mouse and anti-rabbit secondary antibodies (1:1000 dilution) at RT. The primary antibodies used are listed in Supplementary Table S2, each of which was used at a final concentration of 1 μg/mL. Blots were then visualized using a chemiluminescence detection system according to the manufacturer’s instructions (ECL kit; Pierce, Rockford, IL, USA).

### 4.9. ELISA

Quantification of cytokines in AF-MSC-norCM or AF-MSC-hypoCM was performed in triplicate by ELISAs (Ray Biotech Inc., Norcross, GA, USA) according to the manufacturer’s instructions [55]. TGFβ1 and VEGF were analyzed in AF-MSC-norCM or AF-MSC-hypoCM. The optimal density of the color reaction was detected at a wavelength of 450 nm using a chemiluminescence reader. The background signal detected at 450 nm was subtracted from the determined values. Delta values were normalized to the extinction obtained from standard curves, and protein contents were calculated for each sample. Cytokine amounts were normalized to identical numbers of cells per milliliter of medium.

### 4.10. Immunofluorescence (IF)

The undifferentiated and/or differentiated AF-MSCs were subjected to immunofluorescence staining, as previously described [29,56]. The primary antibodies used are listed in Supplementary Table S2. To determine the percentage of cells expressing a given marker protein, at least three fields in any given experiment were photographed and the number of positive cells relative to the total number of DAPI-labeled nuclei was determined.

### 4.11. *In Vitro* Wound Healing Assay

Wound-healing assays were performed in triplicate as previously described [34,35]. Briefly, cells were seeded into six-well dishes at a density of 7 × 10^5^ cells/well. The dishes were cultured as confluent monolayers and were then further synchronized in high-glucose DMEM containing 10% FBS at least for 5 h. The cells were scratched once per well with a 200-μL pipette tip to create an artificial wound. *In vitro* injury was induced by creating linear scratches of 500-μm wide strip of cells [34]. The scratch border was marked with a fine black line immediately after the scraping. Wounded cell cultures were then incubated in the presence of AF-MSC-norCM and AF-MSC-hypoCM for 6–24 h, and others were done in AF-MSC-hypoCM with SB431524 (TGFβ1 inhibitor, 25 μM) or anti-VEGF (2 μg/mL) for 12 h, respectively. The migration of cells was assessed as a function of how far from the scratch line the cells had progressed and the overall number of cells migrating over the 24-h period.

### 4.12. *In Vivo* Wound Healing Assay

ICR mice (8 week-old, female, body weight 20 g) were obtained from The Jackson Laboratory. The animals were randomly divided into three groups (10 mice/group) and the excisional wound splinting model was generated as described previously [17,37]. In brief, after hair removal from the dorsal surface and anesthesia, two 2-mm full thickness excisional skin wounds were created on each side of the midline. Each wound received either AF-MSC-norCM or AF-MSC-hypoCM by both subcutaneous injection around the wound and topical application to the wound bed. A donut-shaped silicone splint was positioned so that the wound was centered within the splint. An immediate-bonding adhesive (Krazy Glue) was used to fix the splint to the skin, followed by interrupted sutures to stabilize its position and the application of Tegaderm (3 M) over the wounds. Digital photographs of the wounds were taken at 0, 4, 6, and 8 days. Time to wound closure was defined as the time at which the wound bed was completely re-epithelialized and was filled with new tissue. The wound area was measured by tracing the wound margin and was calculated using an image analysis program (NIH Image). The percentage of wound closure was calculated as: (area of original wound – area of actual wound)/area of original wound × 100%. The percentage of conditioned medium-induced wound closure mean the value of DMEM/F12 converted to 100. Mice were sacrificed at 8 days, at which time skin samples, including the wound and 2 mm of the surrounding skin, were harvested for histology using a 2 mm punch biopsy. A part of tissue specimens were fixed in 10% freshly prepared formalin for 24 h and were embedded in paraffin for hematoxylin and eosin (H&E) staining [34]. Another part of tissue specimens were fixed in 4% paraformaldehyde for 3,3′-diaminobenzidine tetrahydrochloride (DAB) staining. Four-micron-thick tissue sections were cut using a microtome, and were collected on superfrost plus poly-L-lysin-coated slides. The embedded tissue sections were then rehydrated by gradual immersion in 70%, 80%, 95%, and 100% ethanol, cleared with xylene, and finally mounted in permount solution (Fisher Scientific, Springfield, NJ, USA), and then washed with xylene to remove paraffin prior to H&E staining. Likewise, the slides with paraformaldehyde-fixed tissue sections were incubated with 3% H_2_O_2_ for 10 min to block endogenous peroxidase activity, and then incubated for 60 min at room temperature with a blocking antibody and subsequently for 16–18 h at 4 °C with the primary antibodies (anti-Laminin, anti-Fibronetcin, anti-AKT, anti-PI2K and anti-SMAD2/3, 1:200 dilution, Supplementary Table S2). The slides were washed with PBS, followed by incubation for 1 h at room temperature with second anti-mouse or anti-rabbit antibody (1:400 dilution). After washes with PBS, the slides were incubated for 30 min with horseradish peroxidase and reacted with DAB as chromogen. The images were taken with an Olympus DP70 camera system (Olympus, Tokyo, Japan). This study was conducted in accordance with the guidelines for the care and use of laboratory animals provided by Korea University, and all experimental protocols were approved by the Ethics Committee of Korea University.

### 4.13. Microarray Analysis

For control and test RNAs, the synthesis of target cRNA probes and hybridization were performed using Agilent’s Low RNA Input Linear Amplification kit (Agilent Technology, Palo Alto, CA, USA) according to the manufacturer’s instructions. Briefly, each 1ug total RNA and T7 promoter primer mix and incubated at 65 °C for 10 min. cDNA master mix (5X First strand buffer, 0.1 M DTT, 10 mM dNTP mix, RNase-Out, and MMLV-RT) was prepared and added to the reaction mixer. The samples were incubated at 40 °C for 2 h and then the RT and dsDNA synthesis was terminated by incubating at 65 °C for 15 min. The transcription master mix was prepared as the manufacturer’s protocol (4X Transcription buffer, 0.1 M DTT, NTP mix, 50% PEG, RNase-Out, Inorganic pyrophosphatase, T7-RNA polymerase, and Cyanine 3/5-CTP). Transcription of dsDNA was performed by adding the transcription master mix to the dsDNA reaction samples and incubating at 40 °C for 2 h. Amplified and labeled cRNA was purified on cRNA Cleanup Module (Agilent Technology) according to the manufacturer’s protocol. Labeled cRNA target was quantified using ND-1000 spectrophotometer (NanoDrop Technologies, Inc., Wilmington, DE, USA). After checking labeling efficiency, fragmentation of cRNA was performed by adding 10× blocking agent and 25× fragmentation buffer and incubating at 60 °C for 30 min. The fragmented cRNA was resuspended with 2× hybridization buffer and directly pipetted onto assembled Agilent’s Human Oligo Microarray (44 K) (Agilent Technology). The arrays hybridized at 65 °C for 17 h using Agilent Hybridization oven (Agilent Technology). The hybridized microarrays were washed as the manufacturer’s washing protocol (Agilent Technology).

### 4.14. Data Acquisition and Analysis

The hybridized images were scanned using Agilent’s DNA microarray scanner and quantified with Feature Extraction Software (Agilent Technology, Palo Alto, CA, USA). All data normalization and selection of fold-changed genes were performed using GeneSpringGX 7.3 (Agilent Technology). The averages of normalized ratios were calculated by dividing the average of normalized signal channel intensity by the average of normalized control channel intensity. Functional annotation of genes was performed according to Gene OntologyTM Consortium (http://www.geneontology.org/index.shtml) by GeneSpringGX 7.3. Gene classification was based on searches done by BioCarta (http://www.biocarta.com/), GenMAPP (http://www.genmapp.org/), DAVID (http://david.abcc.ncifcrf.gov/), and Medline databases (http://www.ncbi.nlm.nih.gov/).

### 4.15. Statistical Analysis

All values are expressed as means ± SD. Student’s paired *t*-test was performed for comparison of data of paired samples. Probability (*p*) values of <0.01 and <0.05 were considered significant.

## 5. Conclusions

In summary, our data provide evidence that hypoxia increases the secretion of paracrine factors of AF-MSCs, and that AF-MSC-hypoCM improves wound healing via enhancement of cell migration and activation of TGF-β/SMAD2 and PI3K/AKT pathways, suggesting that AF-MSC-hypoCM can be used in the treatment of wound healing and postoperative scar. Furthermore, AF-MSC-hypoCM may provide an alternative therapeutic option for medical and cosmetic fields to improve the effectiveness of tissue regeneration.

## Supplementary Information



## Figures and Tables

**Table 1.** The significantly regulated genes classfied according to their function under hypoxia (>1.25-fold).

Biological process	Gene Symbol

Glycolysis	*PFKP*, *ALDOC*, *ENO2*, *TPI1P2*, *PGM1*
Response to hypoxia	*VLDLR*, *PLOD1*, *TFRC*, *PLOD2*, *ALDOC*, *BNIP3*, *EGLN1*
Regulation of cellular component movement	*MAP2KQ1*, *HMOX1*, *INSR*
Response to nutrient levels	*VLDLR*, *AQP3*, *LIPG*, *HMOX1*, *INSR*, *SUOX*, *STC2*
Regulation of cell migration	*INSR*, *MAP2K1*, *HMOX1*
Transcription regulator activity	*SAP30*, *RFX2*, *BTG1*, *ID3*, *NKIL3*, *SCAI*, *KLF7*
Cytokines	*VEGFR*, *FLT1*, *IL4*, *IL6*, *IL15*, *IL17A*, *IL32*, *IL33*, *IL2RG*, *IL3RA*, *FGF20*, *FGFR3*, *FGFR2*, *FGF1*
