# Supplementary material for: Hypoxic Conditioned Medium from Human Amniotic Fluid-Derived Mesenchymal Stem Cells Accelerates Skin Wound Healing through TGF-β/SMAD2 and PI3K/Akt Pathways
Source: Int J Mol Sci. 2014 Jan 6;15(1):605–28. doi: 10.3390/ijms15010605 (PMC3907828; doi:10.3390/ijms15010605)
Supplement: Supplementary file 1 [file ijms-15-00605-s001.pdf]

Supplementary Information

Figure S1. The analysis of a microarray data set as a heat map comparing global gene expression patterns among AF-MSCs cultured under hypoxic and normoxic condition.

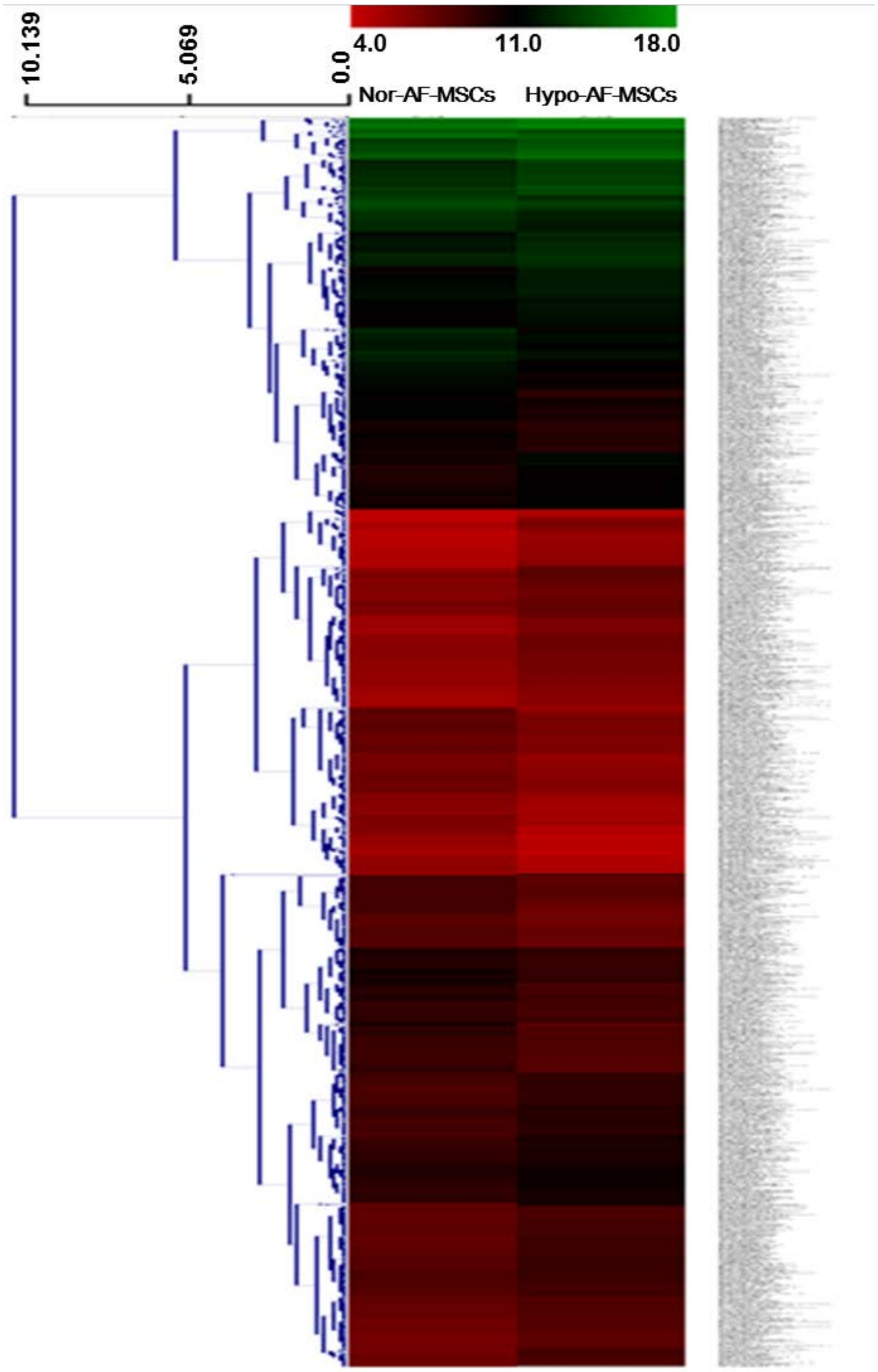

Table S1. Primer sequences used in RT-PCR and real-time PCR.

Gene	Accession No.	Primer sequence (5'-3')	Anealing Temperature (°C)	Product Size (bp)
VEGF	NM_001204385.1	F-CTACCTCCACCATGCCAAGT<br>R-GCAGTAGCTGCGCTGATAGA	62	109
Osteopontin	NM_001040060	F-GAGACCCTTCCAAGTAAGTCCA<br>R-GATGTCCTCGTCTGTAGCATCA	62	354
Osteocalcin	NM_199173	F-GAGCCCCAGTTCCCCTACCC<br>R-GCCTCCTGAAAGCCGATGTG	62	405
LPL2	NM_000237	F-AGAGAGGACTTGGAGATGTGGA<br>R-GGAAGACTTTGTAGGGCATCTG	62	264
aP2	NM_001442	F-AAGAAGTAGGAGTGGGCTTTGC<br>R-CCACCACCAGTTTATCATCCTC	62	285
PPARγ	NM_005037	F-TTGGTGACTTTATGGAGCCC<br>R-CATGTCTGTCTCCGTCTTCTTG	62	311
Collagen 3	NM_000090.3	F-CTGAAATTCTGCCATCCTGAAC<br>R-GGATTGCCGTAGCTAAACTGAA	62	236
Vitronection	NM_000638.3	F-ACGATGGCGAGGAGAAAAAC<br>R-TATTTCGGGGGTAATCAGGG	62	492
Fibronectin	NM_002026.2	F-AAGATTGGAGAGAAGTGGGACC<br>R-GAGCAAATGGCACCGAGATA	62	179
MMP1	NM_002421.2	F-TTGAGAAAGCCTTCCAACTCTG<br>R-CCGCAACACGATGTAAGTTGTA	62	250
Syndecan 2	NM_002998.3	F-CAGAAACCAACAAGTGAGAGGG<br>R-AGCTTCTTCAATGGAGCTGTTG	62	361
Syndecan 4	NM_002999.2	F-CCTAGAAGGCCGATACTTCTCC<br>R-ACCTTGTTGGACACATCCTCAC	62	292
Elastin	NM_000501.1	F-ATCAACGTTGGTGCTACTGCTT<br>R-ATCTTTAGAGGAGCCCCAGGTA	62	362
SPP1	NM_0010400060.1	F-CTGGAAGTTCTGAGGAAAAGCA<br>R-TCAGGGTACTGGATGTCAGGTC	62v	401
GAPDH	NM_002046	F-GTGGTCTCCTCTGACTTCAACA<br>R-CTCTTCCTCTTGTGCTCTTGCT	62	211

Table S2. Antibody used in FACS, western blot, ELIZA, IF, and IHC.

**Antibody**	**Company**	**Host**
CD13	R&D System	Mouse
CD29	BD Bioscience	Mouse
CD31	BD Bioscience	Mouse
CD34	BD Bioscience	Mouse
CD44	BD Bioscience	Mouse
CD90	BD Bioscience	Mouse
p21	Cell Signaling	Mouse
pRb	Cell Signaling	Rabbit
p-Akt	Cell Signaling	Rabbit
Akt	Cell Signaling	Rabbit
PI3K	Cell Signaling	Rabbit
p-MEK	Cell Signaling	Rabbit
MEK	Cell Signaling	Rabbit
p-ERK	Cell Signaling	Rabbit
ERK	Santa Cruz	Goat
p-SMAD2	Cell Signaling	Rabbit
p-SMAD3	Cell Signaling	Rabbit
SMAD2/3	Cell Signaling	Rabbit
HIF1-α	Abcam	Mouse
TGF-β1	Cell signaling	Rabbit
Fibronectin	Abcam	Mouse
Laminin	Millipore	Mouse
β-Actin	Sigma	Rabbit
Tubulin	Sigma	Mouse

© 2014 by the authors; licensee MDPI, Basel, Switzerland. This article is an open access article distributed under the terms and conditions of the Creative Commons Attribution license (http://creativecommons.org/licenses/by/3.0/).
